# The Silent Valve Culprit: A Case Report and Literature Review of Isolated Cabergoline-Induced Mitral Regurgitation

**DOI:** 10.7759/cureus.113803

**Published:** 2026-08-02

**Authors:** Hiba Haouani, Hiba Elbakkali, Najlaa Belharty, Latifa Oukerraj, Mohamed Cherti

**Affiliations:** 1 Department of Cardiology B, Ibn Sina Hospital, Rabat, MAR; 2 Department of Cardiology B, Ibn Sina Hospital, Mohammed V University, Rabat, MAR

**Keywords:** cabergoline, cardiotoxicity, dopamine agonist, drug-induced valvular disease, echocardiography, mitral regurgitation, parkinson’s disease, serotonin receptor

## Abstract

Cabergoline is an ergot-derivative dopamine agonist, widely used in the management of hyperprolactinemia and Parkinson's disease. However, its long-term use is associated with cardiac toxicity linked to its affinity for serotonin receptors present in valve tissue. This serotonin agonist activity can induce valvular heart disease, with a spectrum of severity ranging from mild involvement to severe hemodynamic compromise. We report a case of isolated mitral regurgitation in a 58-year-old male patient with a 12-year history of Parkinson's disease, presenting with inaugural acute decompensated heart failure in the context of previously unrecognized long-term cabergoline exposure. The toxicity manifested after a latency of nine years and significantly impacted quality of life. We additionally conducted a literature review to better elucidate the clinical manifestations, patient outcomes, and other molecules sharing a similar cardiotoxicity profile. This case underscores the need to consider drug-induced valvular heart disease in the differential diagnosis, especially in regions where rheumatic etiology dominates the clinical landscape. Baseline and follow-up echocardiographic evaluations in patients receiving long-term cabergoline therapy are essential for early detection and prevention of irreversible valvular damage.

## Introduction

Parkinson's disease is a progressive neurodegenerative disorder requiring long-term dopaminergic therapy, among which ergot-derived dopamine agonists such as cabergoline are widely used. Beyond their neurological benefits, these agents carry a well-recognized yet frequently underappreciated risk of drug-induced valvular heart disease (DIVHD). Through their affinity for serotonin 5-HT2B receptors, they activate downstream cellular signaling pathways that promote valve leaflet thickening, fibrosis, and retraction [1,2]. This association has been particularly well documented in patients with Parkinson's disease. However, both patients and clinicians often remain unaware of the progressive and insidious nature of this adverse effect, resulting in inadequate echocardiographic surveillance during chronic therapy.

The epidemiological landscape of valvular heart disease in low-to-middle-income countries is largely dominated by rheumatic heart disease, which remains the leading etiology in these settings. This epidemiological dominance means that rarer causes, such as DIVHD, are frequently overlooked or misattributed [3]. We report a rare case of cabergoline-induced isolated mitral regurgitation in a Moroccan patient, likely representing the first locally reported case, and highlight an underrecognized yet clinically significant cardiac complication with potentially serious consequences when diagnosis is delayed.

## Case presentation

A 58-year-old man with a 12-year history of Parkinson's disease, managed with a levodopa-benserazide combination, was referred to our cardiology department for further evaluation of a newly diagnosed severe mitral regurgitation, identified in the context of acute decompensated heart failure. He had no prior cardiac history, no cardiovascular risk factors, no history of acute rheumatic fever, and denied any history of angina or chest pain.

**Figure 1:**
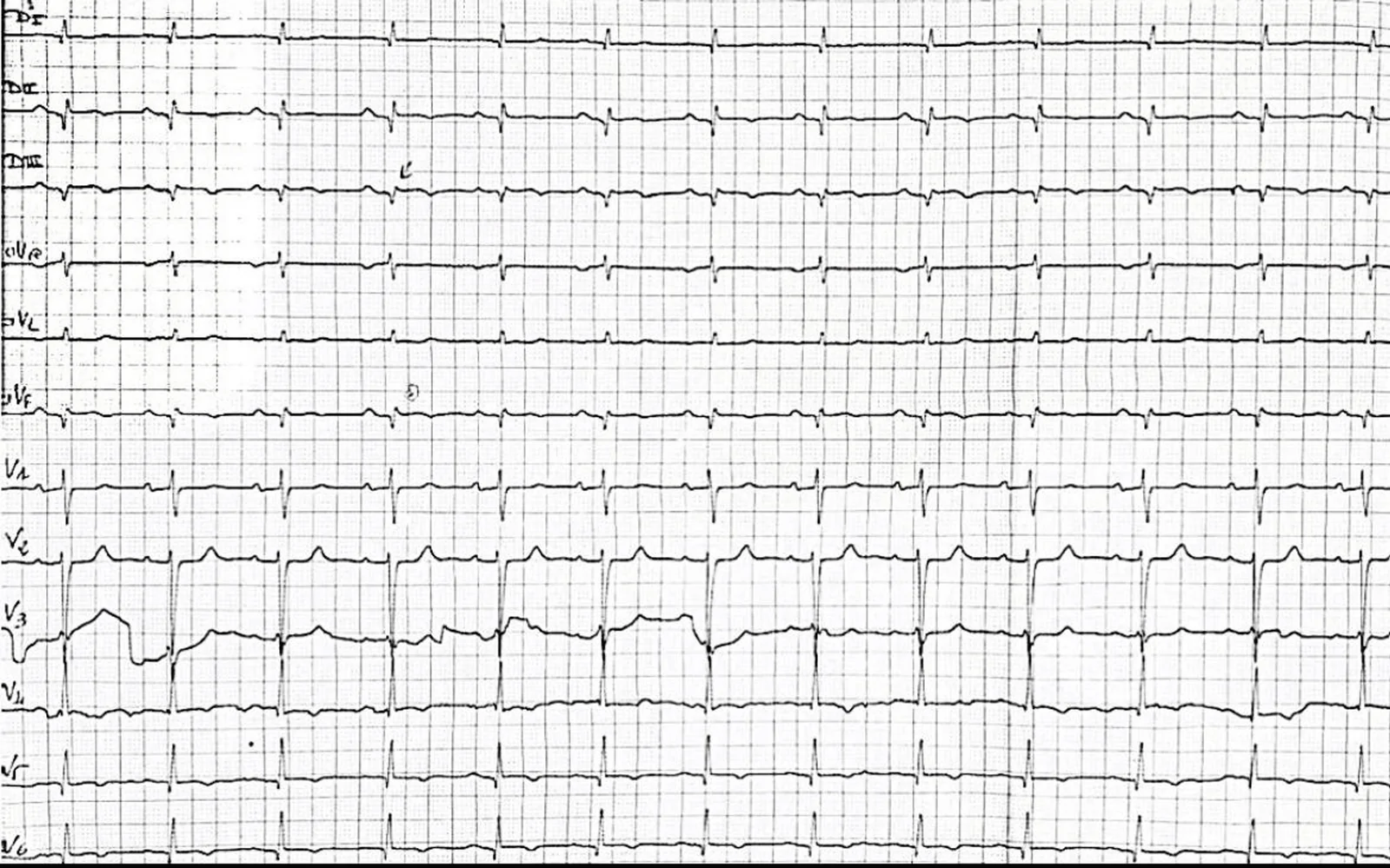
Twelve-lead ECG performed upon admission showing low voltage in limb leads, first-degree AV block and fragmented QRS complexes in the inferior territory. ECG: Electrocardiogram; AV: atrio-ventricular.

One year prior to presentation, the patient developed progressive exertional dyspnea evaluated initially as class II as per the New York Heart Association (NYHA) functional classification [4], which gradually worsened to class III. One week before admission, he presented with acute heart failure, manifesting clinical signs of both left- and right-sided congestion. Electrocardiography on admission (Figure 1) showed sinus rhythm with first-degree atrioventricular block, low-voltage limb leads, and fragmented QRS complexes in inferior leads.

Upon admission, transthoracic echocardiography (TTE) revealed thickened and mildly retracted mitral valve leaflets with restricted mobility, resulting in severe mitral regurgitation (Video 1). The left ventricle was dilated with global hypokinesia and a reduced ejection fraction of 32% (Simpson Biplane method). The left atrium was markedly enlarged, with a left atrial area of 34 cm² and an indexed left atrial volume of 92 mL/m². Doppler parameters were consistent with elevated left ventricular filling pressures. No significant involvement of the aortic, tricuspid, or pulmonary valves was observed. Importantly, there were no calcifications, no chordal shortening, no commissural fusion, and no leaflet prolapse, making diagnosis of rheumatic and degenerative etiologies less likely.

**Video 1:** Three-chamber apical view on TTE showing eccentric regurgitant flow through the mitral valve, with restricted mobility. TTE: Transthoracic echocardiography.

In the presence of left ventricular dilation and systolic dysfunction, coronary angiography was performed to differentiate ischemic cardiomyopathy with secondary mitral regurgitation from primary mitral valve disease contributing to heart failure progression. Coronary angiography (Video 2) revealed no obstructive epicardial coronary artery disease. At this stage, the severity of mitral regurgitation was considered to be partially influenced by elevated filling pressures, suggesting an additional hemodynamic component.

**Video 2:** Coronary angiography showing non-atherosclerotic epicardial coronary arteries.

**Figure 2:**
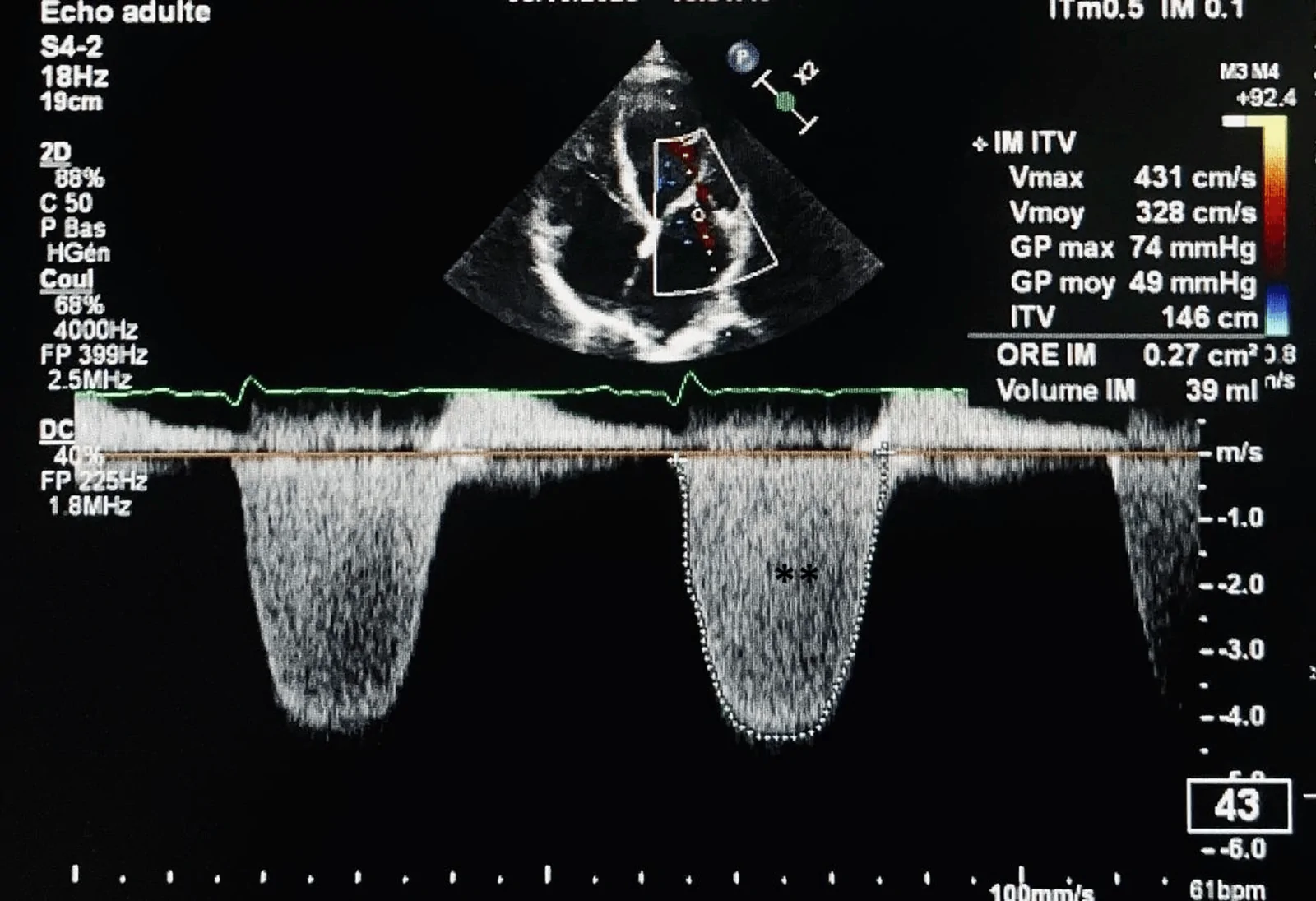
Apical four-chamber view of Doppler assessment of mitral regurgitation, showing on CW Doppler a dense and parabolic jet (**double asterisk). VTI: Volume time integral; CW: continuous wave; mitral VTI/aortic outflow VTI ratio = 1.21.

**Figure 3:**
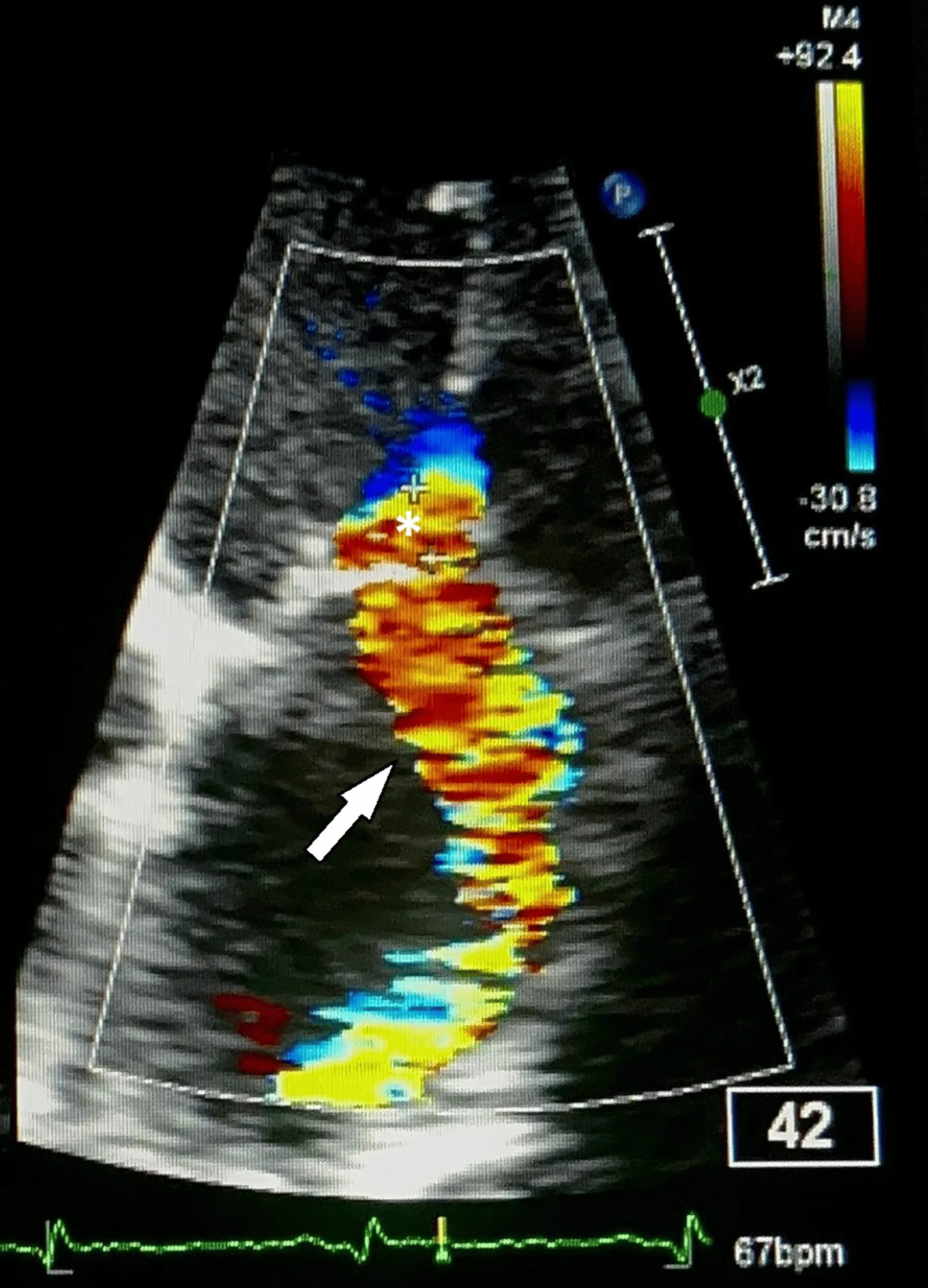
Apical four-chamber color view with focus on trans-mitral flow showing eccentric mitral regurgitation with a posteriorly directed jet (white arrow). MR flow convergence zone (white asterisk) coherent with a Grade III MR. PISA method estimating EROA =0.31 cm², Reg Vol= 45 mL. MR: mitral regurgitation, EROA: effective regurgitant orifice, Reg Vol: regurgitant volume.

Following diuretic therapy and hemodynamic optimization, a second TTE demonstrated persistently thickened mitral leaflets with systolic and diastolic restriction of the posterior mitral leaflet. The regurgitant jet was eccentric, coursing along the atrial surface of the posterior leaflet (Video 1, Figure 3). Quantitative assessment was consistent with moderate-to-severe organic-functional mitral regurgitation (Figure 2, Figure 3). Cardiac magnetic resonance imaging confirmed diffuse myocardial fibrosis without identifying an alternative etiology, further supporting a primary valvular origin.

A thorough retrospective review of the patient's medical records revealed a history of long-term cabergoline exposure at a dose of 1 mg/day, initiated nine years before symptom onset, and maintained for approximately three years, corresponding to a cumulative dose of 1,095 mg. In the absence of any other plausible etiology, and given the well-established association between cabergoline and fibrotic valvular disease, a diagnosis of cabergoline-induced mitral valvulopathy was strongly suspected.

Following initiation of guideline-directed medical therapy for heart failure, the patient's symptoms stabilized at follow-up. A letter was addressed to the treating neurologist, emphasizing the need for cabergoline discontinuation and recommending ongoing cardiac surveillance given the risk of further valvular fibrosis progression and potential hemodynamic deterioration.

## Discussion

The mechanisms underlying mitral regurgitation in our case are both organic and functional, with the primary and predominant contribution being organic, manifesting as valve leaflet thickening with systolic and diastolic restricted mobility, consistent with a Carpentier type IIIA mechanism. Secondary mitral annular dilation, resulting from progressive left atrial and left ventricular enlargement, added a Carpentier type I component. The combination of both mechanisms explains the progressive nature of the regurgitation observed in our patient.

The diagnosis of rheumatic mitral valve disease was considered but excluded. Characteristic echocardiographic features of rheumatic disease, including commissural fusion, chordal shortening, and subvalvular apparatus involvement, were notably absent. Furthermore, no other valve was morphologically affected, making rheumatic origin unlikely. Degenerative etiology was similarly dismissed given the absence of leaflet prolapse, excess tissue, and chordal elongation. Ischemic cardiomyopathy with secondary mitral regurgitation was ruled out by coronary angiography, which revealed no obstructive epicardial coronary artery disease, and further confirmed by cardiac magnetic resonance imaging. In the absence of any alternative etiology, and given the characteristic echocardiographic morphology of leaflet disease, a diagnosis of DIVHD secondary to cabergoline therapy was strongly suspected.

**Figure 4:**
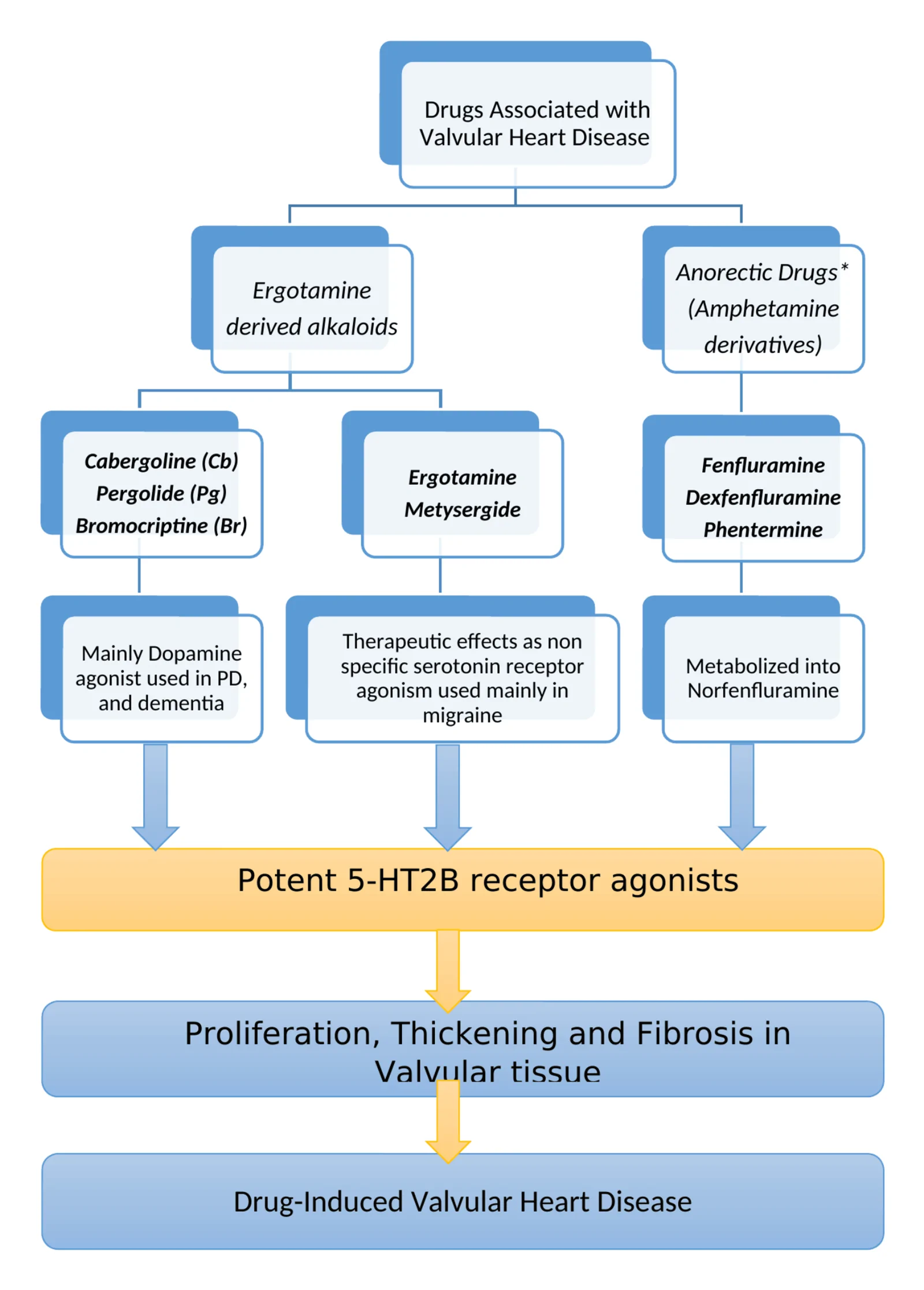
Schematic representation of serotonin-mediated pathway leading to DIHVD. DIVHD: Drug-induced valvular heart disease; *: withdrawn by the Food and Drug Administration; PD: Parkinson's disease. Created by the authors using the Smart Art option and Forms options in Microsoft Word

Ergot-derived dopamine agonists, including cabergoline and pergolide, are well-established causes of DIVHD, specifically producing restrictive valvular disease through impaired leaflet coaptation and subsequent regurgitation. These agents exert their cardiotoxic effects through agonist activity at serotonin 5-HT2B receptors, which are highly expressed on the surface of valvular interstitial fibroblasts. Receptor activation triggers downstream mitogenic signaling pathways, promoting myofibroblast proliferation and extracellular matrix remodeling, with a net result of non-calcific fibrotic thickening and retraction of valve leaflets. These molecular and histological findings have been extensively documented in both clinical series and experimental animal models [5,6]. Notably, non-ergot-derived anorectic agents share an identical cardiotoxicity profile through the same serotonin-mediated mitogenic pathway, underscoring serotonin as the central mediator of this form of DIVHD [7]. A simplified diagram illustrating this toxicity pathway is proposed in Figure 4. Other pharmacological agents used in Parkinson's disease management, such as levodopa, have been associated with increased cardiovascular risk and heart failure; however, they do not directly induce valvular fibrosis in the manner characteristic of ergot-derived molecules [8].

Our case illustrates an isolated mitral regurgitation with an nine-year latency period following cabergoline exposure, and contributes to the growing body of literature demonstrating that drug-induced valve disease may occur outside the classical setting of high-dose therapy in Parkinson's disease populations. To further elucidate the clinical characteristics and outcomes of this condition, we conducted a literature review of all reported cases in the English literature where valvular heart disease resulted from drug-induced toxicity.

Literature search methodology

Our literature search was performed using the PubMed database for articles published up to February 2026. Search keywords included "heart valve disease," "dopamine agonists," "cabergoline," "bromocriptine," "ergotamine," and "pergolide." We manually scanned relevant studies to identify case reports and patients from case series meeting our inclusion criteria, using the following search equation: (ergotamine OR dopamine agonists OR cabergoline OR bromocriptine OR pergolide) AND (heart valve disease). Including our case, clinical data from 71 patients with ergot-derived drug or anorectic agent-induced valve disease are summarized in Table 1.

**Table 1:** Review of published cases of Serotonin-receptor-agonist mediated valve-disease. **: cases from Case series, or Case studies, YO: years old, F: female, M: male, YO: Year old, CAV: cabergoline-associated valvulopathy, Cb: cabergoline, Br: bromocriptine, Pg: pergolide (high doses of Pg : sup to 5mg daily), Fen: fenfluramine, DexFen: dexfenfluramine, Erg: ergotamine, Phen: phentermine, HF: heart failure, MR: mitral regurgitation, MS: mitral stenosis, AR: aortic regurgitation, TR: tricuspid regugitation, TS: tricuspid stenosis, PR: pulmonary regurgitation, PHT : pulmonary hypertension, NS : not specified, PD: Parkinson’s disease, ACE: angiotensin-converting-enzymes, AVR: aortic-valve replacement, MVR: mitral-valve replacement, Tid: three times a day.

Study	Age and Sex	Affected valves	Severity of valvular disease/TTE findings	Ergot derived drug-type	Drug indication	Treatment duration	Dose	Other contributing mechanism in Valvular disease	Follow up and evolution	Pathological Findings/ Miscellaneous
Connolly et al (1997) [9] **	41 YO/ F	Mitral	Severe MR -Thickened MV	-Phen -Fen	Obesity treatment (Appetite suppressants)	25 mo	Phen: 48mg a day Fen: 120mg a day	Ruled out	MV repair	Glistening white, thickened leaflets
	44 YO/F	Aortic -Mitral -Tricuspid	Severe AR -Severe MR			12 mo	Phen: 30 mg a day Fen: 60mg a day		AVR, MVR, tricuspid valve repair	No chordae rupture or flail segment, aortic valve with a “stuck-on” appearance of plaque on leaflets
	48 YO/F	Mitral -Aortic	Severe MR -Moderate AR			9 mo	Phen: 30mg a day Fen: 60mg a day		MVR	No prolapse or chordal rupture, with a “stuck-on” appearance of plaque on leaflets
	52 YO/F	Mitral	Severe MR			12 mo	Phen: 15mg a day Fen: 40mg a day		MV repair	Thickened and tethered posterior leaflet.
	49 YO/F	Mitral	Severe MR			11 mo	Phen: 30mg a day Fen: 60mg a day		MVR	Glistening white, thickened leaflets and chordae.
	51 YO/F	Aortic -Mitral -Tricuspid	Moderate AR -Moderate TR -Severe MR			7 mo	Phen: 30mg a day Fen: 60mg a day		Medical treatment of HF: Phen and Fen discontinuation, with stable evolution.	NS
	44 YO/F	Aortic	Moderate AR			12 mo	Phen: 30mg a day Fen: 60mg a day		NS	NS
	41 YO/F	Mitral -Tricuspid -Aortic	Severe TR -Severe MR -Moderate AR	-Ph -Fe		6 mo	Ph: 30mg a day Fe: 60mg a day		NS	NS
	50 YO/F	Mitral	Moderate MR		Obesity treatment	4 mo	Ph: 30mg a day Fe: 40mg a day	Ruled out	NS	NS
	50 YO/F	Aortic -Mitral	Moderate AR -Mild MR			6 mo	Phen: 15mg a day Fen: 40mg a day	Ruled out	NS	NS
	42 YO/F	Aortic -Mitral -Tricuspid	Moderate AR -Severe MR -Severe TR			1 mo	Phen: 30mg a day Fen: 60mg a day	Ruled out	NS	NS
	41 YO/F	Mitral -Tricuspid -Aortic	Moderate MR -Moderate TR -Moderate AR			6 mo	Phen: 30mg a day Fen: 40mg a day	Ruled out	NS	NS
	48 YO/F	Mitral -Aortic -Tricuspid	Severe MR -Moderate AR -Moderate TR			15 mo	Phen: 30mg a day Fen: 20mg a day	Ruled out	NS	NS
	34 YO/F	Mitral -Aortic -Tricuspid	Severe MR -Moderate AR -Moderate TR	-Phen -Fen	Obesity treatment	15 mo	Phen: 30mg a day Fen: 60mg a day	Ruled out	NS	NS
	35 YO/F	Aortic -Mitral	Moderate AR -Mild MR			14 mo	Phen: 30mg a day Fen: 40mg a day	Ruled out	NS	NS
	63 YO/F	Aortic -Mitral -Tricuspid	Moderate AR -Moderate TR -Moderate MR			8 mo	Phen: 30mg a day Fen: 60mg a day	Ruled out	NS	NS
	38 YO/F	Aortic -Mitral	Moderate AR -Mild MR			6 mo	Phen: 30mg a day Fen: 40mg a day	Ruled out	NS	NS
	43 YO/F	Aortic -Mitral -Tricuspid	Moderate AR -Mild MR -Mild TR			28 mo	Phen: 30mg a day Fen: 20mg a day	Ruled out	NS	NS
	56 YO/F	Mitral -Tricuspid	Mild MR -Mild TR	-Phen -Fen	Obesity treatment	17 mo	Phen: 30mg a day Fen: 40mg a day	Ruled out	NS	NS
	44YO/F	Aortic	Moderate AR			17 mo	Phen: 30mg a day Fen: 40mg a day	Ruled out	NS	NS
	41 YO/F	Mitral -Tricuspid -Aortic	Mild MR -Mild TR -Mild AR			7 mo	Phen: 30mg a day Fen: 60mg a day	Ruled out	NS	NS
	33YO/F	Mitral -Tricuspid -Aortic	Mild MR -Mild TR -Mild AR			2 mo	Phen: 30mg a day Fen: 40mg a day	Ruled out	NS	NS
	30 YO/F	Aortic -Mitral	Severe AR -Severe MR	-Phen -Fen	Obesity treatment	20 mo	Phen: 30mg a day Fen: 60mg a day	Ruled out	NS	NS
	38 YO/F	Aortic	Mild AR			4 mo	Phen : 30mg a day Fen: 40mg a day	Ruled out	NS	NS
Wilke et al. (1997) [10]	45 YO/F	Mitral -Tricuspid -Aortic	Grade 4 MR -TR and TS -Grade 3 AR	Erg	Migraine	More than 5 years	Up to 10mg a day	Ruled out	Triple Valve replacement	The patient also developed leg gangrene from ergotamine abuse.
Serratrice et al. (2002) [11]	63 YO/M	Tricuspid -Aortic -Mitral	Severe TR with restricted mobility -Mild MR -Mild AR -All valve leaflets were thickened with restricted mobility.	Br	PD	5 years	40 mg a day	Ruled out	Valve disease stabilization upon Bromocriptine withdrawal.	NS
Pritchett et al. (2002) [12]**	72 YO/F	Tricuspid -Aortic	Severe TR with thickened and retracted leaflets -Mild MR	Pg	PD	4 years	3mg a day for 2 years - 6mg a day for 2 years	Ruled out	Tricuspid valve replacement with Pergolide discontinuation.	NS
	74 YO/F	Tricuspid -Mitral -Aortic	Severe TR -Mild TS -Moderate to severe MR and mild MS -Thickened leaflets	Pg	Restless leg syndrome	3 years	3mg a day	Ruled out	Tricuspid, Mitral and aortic valve replacement -Postoperatively uneventful course	NS
	61 YO/F	Tricuspid -Mitral -Aortic	Thickened leaflets -Severe TR -Moderate AR -Mild MR.	Pg	PD	7yeras	3.75 mg daily.	Ruled out	Stable evolution under medical treatment only (asymptomatic patient).	NS
Van Camp et al. (2003) [13] **	61 YO/M	Mitral -Aortic	MR grade III to IV -Mild AR.	-Br for 2 years - Pg for 6months	PD	2years and 6months	5mg daily then switch to Pg 8mg daily	Ruled out	Valve surgery was scheduled, but the patient died suddenly before surgery.	NS
	73 YO/F	Mitral -Aortic -Tricuspid	Severe MR -Moderate to severe AR - Secondary Moderate TR	Br Pg	PD	Br for 1 year Pg for 3 years	1.5 mg of Pg daily for 3 years, then 7mg daily	Ruled out	Bioprosthetic mitral and aortic valve replacement and tricuspid annuloplasty with good outcome.	NS
	71 YO/F	Mitral -Tricuspid	-Grade 1 MR -Grade 1 TR	Pg	PD	14 months	8mg daily of Pg	Ruled out	NS	NS
	71 YO/M	Tricuspid	-Grade 1 TR	Pg	PD	11 months	5mg daily	Ruled out	NS	NS
	69 YO/M	Aortic -Tricuspid	-Grade 2 AR -Grade 2 TR	Pg Br	PD	7 months	6mg daily	Ruled out	NS	NS
	71 YO/M	Mitral	Grade 1 MR	Pg	PD	9 months	6mg daily	Ruled out	NS	NS
	66 YO/F	Mitral	Grade 2 MR	Pg	PD	4 months	5mg daily	Ruled out	Cessation of Pergolide with total regression of MR at 3months.	NS
	81 YO/F	Mitral	Grade 1 MR	Pg	PD	22 months	5 mg daily	Ruled out	NS	NS
	70 YO/ M	Mitral -Aortic	Grade 1 MR -Grade 2 AR	Pg	PD	15 months	6mg daily	Ruled out	NS	NS
	72 YO/ M	Mitral -Aortic -Tricuspid	Grade 2 MR -Grade 1 AR -Grade 2 TR	Pg	PD	11 months	9mg daily	Ruled out	NS	
Goujon-Dubois et al. (2003) [14]	48 YO/F	Tricuspid -Aortic -Mitral	Grade 3 to 4 TR -Grade 2 to 3 AR -Grade 2 MR	Erg	Migraine	28 years	2mg to 4 a day	Ruled out	Multiple valve replacement with favorable outcomes.	Ergotamine-induced valvulopathy was confirmed on histopathological findings of resected leaflets
Calomne et al. (2004) [15]	67 YO/F	Aortic -Mitral	Grade II to III AR -Grade III MR -PTH	Br - Pg	PD	1year of Br and 1 year of Pg	Br 10mg t.i.d -Pg 6mg a day	Ruled out	Pergolide discontinuation with favorable outcome and stabilization.	NS
Horvath et al. (2004) [16]**	58 YO/F	Multi-valvular (4 valves).	Severe MR, -Moderate to severe AR and TR, - Mild PR - Retraction and thickening of these valves	Cb	PD	A total of 20 months with 4mg daily for 7months.	1mg a day then increased to 4mg a day over time and gradually	Ruled out	Discontinuation of Cabergoline with slow improvement and no residual cardiac symptom over time.	NS
	42 YO/F	Multi-valvular	Severe TR with mild TS -Moderate AR -Mild MR	-Br then -Pg	PD	A total of 8 years on ergot-derived drugs.	-20mg a day then 30mg a day of Br -2.5mg t.i.d than 1.5mg t.i.d of Pg.	Ruled out; TTE resembling carcinoid syndrome-related valvulopathy findings	Discontinuation of pergolide, ACE inhibitors, and diuretics treatment with stable clinical evolution.	NS
	74 YO/M	Mitral valve. -Tricuspid valve.	Severe TR -Mild MR	Pg	PD	4 years	3mg a day for 1 year, then 4mg a day for 3years	Ruled out	Tricuspid valve replacement with drug discontinuation and favorable outcome.	NS
	57 YO/F	Tricuspid -Aortic -Mitral	Severe TR -Moderate AR -Moderate MR with all 3 valves retracted and thickened.	Pg	PD	16 months	4.5mg a day	Ruled out	Successful multiple valve repair with mitral annuloplasty without pergolide discontinuation. -Months after surgery, massive HF appeared with improvement on diuretics and ACE inhibitors and pergolide discontinuation.	NS
Roth et al. (2005) [17]	56 YO/F	Multi-valvular disease.	Multi-valvular insufficiency with predominant severe MR.	Pg	PD	18 months	3mg a day	Ruled out	Mitral valve replacement and change to a non-ergotamine-derived drug with favorable stable evolution.	NS
Van Strater et al. (2005) [18]	58YO/F	Tricuspid -Aortic -Mitral valves.	NR	Pg	PD	10 years	NS	Ruled out	Aortic and Tricuspid valve mechanical replacement, with a switch to non-ergotamine-derived drug. -Stable evolution.	Pergolide-related fibro-proliferative abnormalities of the tricuspid, aortic and mitral valves
Pinero et al. (2005) [19]	74 YO/M	Mitral valve	Severe MR through restrictive mechanism (retracted leaflets with incomplete coaptation).	Cb	PD	4 months	NR	Ruled out	Reversible mitral disease upon cabergoline discontinuation but HF symptoms persisted -Mechanical MV replacement was followed with good clinical outcome and HF symptoms regression.	The histopathology of resected leaflets showed myofibroblast proliferation within a fibromyxoid stroma
Bijl et al.(2005) [20]	67 YO/F	Prior aortic valve involvement (Aortic prosthesis) -Mitral -Pulmonary -Tricuspid	Normal aortic prosthesis but thickened mitral, pulmonary, and tricuspid valves, all three severely insufficient.	Erg	Migraines	NS	2mg daily	Ruled out	Mechanical valve replacement of mitral, tricuspid, and pulmonary valves with uneventful recovery and significant symptom improvement.	The pathology examination of leaflets was compatible with long-term use of ergotamine. Previous pathology of aortic valve was consistent with ergotamine use.
Chung et al. (2006) [21]	61 YO/M	Mitral -Aortic -Tricuspid	Severe MR and rupture of chordae tendinae of anterior leaflet. -Mild AR. -Moderate TR.	Pg	PD	9 years	Low daily dose and low cumulative dose of 2730mg	NS	Mitral Valve replacement. -Upon pergolide discontinuation, clinical improvement was evident.	Fibromyxoid valvulopathy without calcification.
Hanada et al. (2007) [22]	82 YO/M	Mitral Valve	Severe MR through retracted leaflets and incomplete coaptation.	Cb	PD	4 years and 8months	NS	Myxoid degeneration.	NS	Histologic analysis showed fibrous thickened mitral chordae with myxoid degeneration
Martin et al. (2007) [23]	49 YO/F	Mitral Valve	Restrictive posterior mitral leaflet and mild to moderate MR.	Cb	PD	10 months	5mg a day	Ruled out	NS	NS
Worthington et al. (2008) [24]	49 YO/F	Mitral -Aortic	-Moderate to severe MR. -Mitral valve stenosis -Moderate AR. -“Hockey-stick” appearance on TTE of mitral leaflets	Pg	Restless leg syndrome	5 years	1.14g cumulative dose, low-dose regimen.	Initially rheumatic origin was suggested, but refuted on histopathological examination of resected valves.	Mechanical aortic and mitral valve replacement -Patient was so incapacitated by the restless-leg syndrome she didn’t stop Pergolide.	No calcification and no neo-vascularization, with a fibrotic and the and retracted posterior leaflet.
Lyons et al. (2008) [25]	57 YO/M	Isolated Aortic valvulopathy	Moderate AR -Tethered leaflets with failure to coaptation.	Cb	Lewy body dementia	12 months	NS	Ruled out	NS	NS
Cawood et al. (2009) [26]	59 YO/M	Mitral valve	Restrictive valvulopathy; thickened mitral leaflets and little involvement of sub-valvular apparatus -Increased mitral tenting area - Moderate functional TR	Cb	Macro-prolactinoma	2 years	0.5mg twice a week then increased to 1.25mg thrice a week: 252 mg of cumulative dose (Low dose).	Ruled out	Mitral valve replacement and tricuspid annuloplasty as the patient developed acute HF over years. -The patient died of Bowel obstruction secondary to an obstructing mass.	Fibro-proliferative tissue on microscopic examination with no other features of calcifications, myxoid origin or inflammation
Chagué et al. (2009) [27]	53 YO/F	Aortic valve	AR with restricted mobility of the thickened cusps.	Fen -DexFen -Br -Cb	Hyper-prolactinemia	-2 months of Fe -13 months of DexFen -Br for 7 years -Cb for 6 years	Cb: 140mg of cumulative dose.	Ruled out	Stable clinical and imaging evolution upon cabergoline withdrawal.	NS.
Apostolakis et al. (2009) [28]	68 YO/M	Aortic -Mitral	Initially moderate aortic and mitral regurgitation that evolved to severe regurgitation.	Pg	PD	3 years	2mg/day	Ruled out	Double valve replacement with ascending aorta replacement with significant improvement.	Renal function deterioration secondary to retroperitoneal fibrosis caused by ergotamine toxicity
Luedde et al. (2009) [29]	75 YO/F	Mitral -Aortic	Severe MR	Cb	PD	6 years	4mg daily	None (other causes ruled out on autopsy).	Fatal cardiogenic shock despite resuscitation, IABP and urgent surgery.	Histopathological changes consistent with fibrous proliferation in leaflets and chordae tendinae
Izgi et al. (2010) [30]	Young/F	Tricuspid	Severe TR	Cb	Acromegaly	1year	0.5 mg/day (low dose)	Ruled out	NS	NS
Bhat et al. (2011) [31]	60 YO/F	Tricuspid	Moderate TR. -Mild PHT	Cb	Micro-prolactinoma	4 years	Low dose: 0.25 mg a day	Ruled out	Stabilization upon medical treatment on Spironolactone and furosemide.	NS
Levin et al. (2011) [32]	51 YO/M	Aortic -Mitral	Grade II MR -Grade II AR -Antero-medial mitral leaflet prolapse	Cb	PD	20 months	Cumulative dose of 8,353 mg	Ruled out	Improvement of symptoms over time with cabergoline discontinuation.	NS
Bois et al. (2012) [33]	55 YO/F	Mitral -NB: Unusual manifestation: Chylothorax.	Severe MR -Sclerotic mitral valve with immobile posterior leaflet -Thickened anterior leaflet - Secondary Moderate tricuspid regurgitation	Erg	Recurrent migraines	34 years	High doses	Ruled out	Mitral valve replacement with stable moderate MR without stenosis. -Significant improvement at 10 years post-operatively.	On gross examination of resected valves: Diffuse leaflet thickening and commissural fusion, shortening of chordae
D’Aloia et al. (2013) [34]	56 YO/M	Mitral	Severe MR with secondary TR	Cb	Prolactinoma	6 months	0.5mg once daily	Ruled out	-Mitral replacement with good outcomes	NS
Lazopoulos et al. (2013) [2]	54 YO/F	Aortic -Tricuspid -Mitral	Severe MR, with leaflet thickening and restriction. -Moderate AR -Moderate TR	Erg	Migraine	More than 15 years on self-medication.	2 to 4mg /day	Ruled out	Mitral and aortic valve replacement with mechanical prosthesis -Tricuspid valve annuloplasty with good outcomes.	Histo-pathological findings of resected valve leaflets found sub-endothelial proliferation of myofibroblast-like cells positive for smooth muscle actin marker
Patel et al. (2013) [35]	47 YO/F	Mitral -Aortic	Thickened leaflets of mitral and aortic valves with: Restricted valve mobility and cups doming causing moderate mitral stenosis Severe MR -Severe AR. Secondary Severe PHT	Erg	Severe Migraines	10 years	NS	Ruled out	Uneventful Mechanical Mitral and aortic valve replacement	Pathology of resected valves found tan-white, thickened tissue with fibrotic nodules without calcification, with mucoid substance and elastic fibers.
Cautres et al. (2014) [36]	76 YO/M	Aortic, -Tricuspid -Mitral	Thickened leaflets with drumstick appearance and restrictive motion of the two mitral leaflets causing severe MR. Restrictive motion of the right and the non-coronary aortic cusps leading to significant AR. Significant TR with thickened tricuspid leaflets and a dilated tricuspid annulus.	Br	PD	7 years	30mg /day (cumulative dose of 77g)	No significant other cause was deemed contributive.	The patient was managed medically. Br treatment was interrupted.	NS
Maréchaux et al. (2015) [37]	67 YO/F	Mitral	Mild to moderate MR -Leaflet thickening -Retraction towards the ventricular apex causing leaflet tenting and reduced valve mobility Thickening and shortening of chordae tendineae.	Erg (Gynergene)	Migraine	30 years	1 to 3mg	No other mechanism was found.	Evolved to severe MR -Clinical improvement at 2-year follow-up, with furosemide and enalapril.	NS
Mohan et al. (2017) [38]	27 YO/M	Tricuspid	Moderately severe TR	Cb	Prolactinoma	9 months	0.5mg twice a week (low cumulative dose)	Ruled out	Favorable evolution after drug discontinuation.	NS
Caputo et al. (2018) [39]	52 YO/F	Aortic	Moderate to severe AR, with thickened and restricted valve consistent with CAV.	Br followed by Cb.	Macro-prolactinoma	25 years	Cumulative dose of 4192 mg of Cb (3mg a week).	Ruled out	With Cabergoline discontinuation: progression to moderate to severe aortic regurgitation.	NS
Tessier et al. (2023) [40]	60 YO/M	Aortic -Mitral.	Aortic and mitral valve thickening and regurgitation	Br then switch to Cb	Hyper-prolactinemia	5 years of Br -15 years of Cb	649mg of cabergoline (considered as low cumulative dose)	Ruled out	NS	NS
Present Case (2025)	58 YO/M	Mitral	Moderate MR	Cb	PD	3years	Cumulative dose of 1095mg (1mg a day)	Ruled out	Asymptomatic on HF treatment only.	NS

Main indications

In the reported cases, ergotamine derivatives and anorectic agents were used across a broad range of indications, including Parkinson's disease, hyperprolactinemia, acromegaly, recurrent migraines, restless leg syndrome, dementia, and obesity management. Alternative causes of valvular disease were not identified in most cases, and in a subset, a causative relationship was confirmed on pathological examination.

Parkinson's disease was the most frequent indication for ergot-derived drug use in the reviewed cases, followed by obesity treatment. Notably, 23 of the 71 reported cases were attributable to appetite suppressants, representing the second most common indication. Although the Food and Drug Administration (FDA) has since restricted these agents to narrow indications in pediatric epilepsy syndromes, the cases reported in 1997 preceded these regulatory changes and highlight the significant cardiotoxic potential of this drug class [41].

Endocrine disorders accounted for eight cases, with alkaloid derivatives prescribed for hyperprolactinemia generally at lower doses than those used in Parkinson's disease. Migraines accounted for seven cases and restless leg syndrome for two. It is worth noting that self-medication with ergotamine derivatives for migraine management is not uncommon, and thorough drug history taking is essential, as resulting valve disease may be severe enough to necessitate multiple surgical valve repairs [2]. Among dopamine agonists, pergolide was the most frequently implicated agent, followed by cabergoline. Heart failure signs and symptoms represented the predominant clinical manifestation across reported cases of this form of DIVHD.

Valve involvement pattern

Despite sharing a common serotonin-mediated pathway, the disease manifests with considerable variability in terms of valve involvement. Based on our review, the regurgitant form predominates, with 122 instances of valvular regurgitation reported across 71 patients, reflecting the frequent occurrence of multi-valvular disease. Of these, 34 were graded as severe. Valvular stenosis was rarely observed, with only four reported cases. Multi-valvular involvement affecting three valves simultaneously was the most frequent pattern, occurring as commonly as isolated valve disease, underscoring the severity potential of this form of drug toxicity. Simultaneous involvement of all four valves was reported in a single exceptional case. Anatomically, the mitral valve was the most frequently affected, followed by the tricuspid valve [9-40]. Our case represents an example of isolated mitral valve regurgitation.

Echocardiographic features

Clinicians should be familiar with the typical echocardiographic features of ergot-derived drug-induced restrictive valvular disease to facilitate early recognition. Characteristic findings include restricted leaflet mobility, leaflet thickening and tenting, absence of calcification, and absence of commissural fusion, collectively conferring a "hockey-stick" appearance to affected valves. These morphological features are diagnostically valuable as they allow clinical suspicion to be raised based on transthoracic echocardiography alone. Valve prolapse is generally not observed, having been exceptionally reported in the mitral valve in only one case [16].

Histopathological findings

Consistent histopathological findings across different valve locations and different implicated molecules reflect the shared underlying mechanism of 5-HT2B receptor activation. On pathological examination, resected valves demonstrate sub-endothelial proliferation of myofibroblast-like cells positive for smooth muscle actin, conferring a glistening white appearance on gross examination. Valves and occasionally the subvalvular apparatus are diffusely thickened, though the overall structure is preserved. Chordae may be shortened. Fibrotic nodules without calcification, containing mucoid substance and elastic fibers, are characteristic findings. Neither perforation nor rupture of the valvular apparatus has been described [2,9,22,33,35,42]. These pathological features closely resemble those of carcinoid heart disease, reflecting the shared serotonin-mediated fibrogenic pathway [4,24,43].

Reversibility and clinical outcomes

Treatment or replacement of one affected valve does not confer protection against future involvement of other valves if drug exposure is not discontinued, as illustrated by the case of a patient maintained on ergotamine who ultimately developed four-valve disease requiring placement of four mechanical prostheses, resulting in significant thrombotic risk [21]. Complete disease reversibility following drug withdrawal alone was exceptionally reported in one case where pergolide exposure lasted only four months [13]. In the majority of cases, drug discontinuation allows for stabilization of valve disease without further progression [44]. However, surgical repair remains necessary in most symptomatic cases, with generally favorable postoperative outcomes. Notably, cabergoline-induced severe mitral regurgitation resulted in fatal cardiogenic shock in one reported patient despite urgent surgical intervention [29], emphasizing the potentially life-threatening nature of this condition and the critical importance of early diagnosis and long-term surveillance.

Dose-effect relationship

A dose-response relationship has been reported but remains a subject of ongoing debate. In Parkinson's disease patients, the risk of valvular regurgitation has been shown to be dose-dependent and higher than in patients treated for hyperprolactinemia, who typically receive lower doses [5]. However, several patients on low-dose regimens have demonstrated valve disease profiles comparable to those on higher doses [5]. Cawood et al. reported a case of severe valvular disease induced by a low-dose cabergoline regimen [26], suggesting that individual susceptibility to the drug's mitogenic effect may play an important and underappreciated role.

## Conclusions

Cabergoline-induced valvular heart disease remains an under-recognized yet clinically significant complication of long-term dopamine agonist therapy. Distinguishing drug-induced valvular lesions from other etiologies, including rheumatic disease, age-related sclerosis, and myxomatous valve disease, is essential to determine whether drug discontinuation is warranted and to prevent progression to more severe and potentially irreversible forms. This underscores the critical importance of thorough medication history taking in any patient presenting with valvular pathology of unclear origin. Cardiologists should equally be familiar with the characteristic morphological features of DIVHD on echocardiography, enabling this diagnosis to be considered and recognized when encountered.

Awareness must be raised not only among patients on self-medication, but across all prescribing specialties, regarding the necessity of close and structured cardiac follow-up. Baseline echocardiography prior to treatment initiation, followed by periodic clinical and echocardiographic monitoring at six to twelve-month intervals, is essential in patients receiving long-term cabergoline therapy, enabling early detection and timely intervention before irreversible valvular damage occurs. While dopamine aims to heal the brain, serotonin scars the heart.
